# Cluster Analysis and Cluster Ranking for Asthma Inpatient Hospitalizations Among Children, Adolescents, and Adults Aged 0 to 19 Years in Cook County, Illinois, 2011–2014

**DOI:** 10.5888/pcd17.190265

**Published:** 2020-01-16

**Authors:** Katie Labgold, Amanda C. Bennett, Kristen M. Wells

**Affiliations:** 1 Department of Public Health Sciences, University of Virginia, Charlottesville, Virginia; 2 Department of Epidemiology, Rollins School of Public Health, Emory University, Atlanta, Georgia; 3 Division of Reproductive Health, Centers for Disease Control and Prevention, Atlanta, Georgia; 4 Office of Women’s Health and Family Services, Illinois Department of Public Health, Chicago, Illinois

**Figure:**
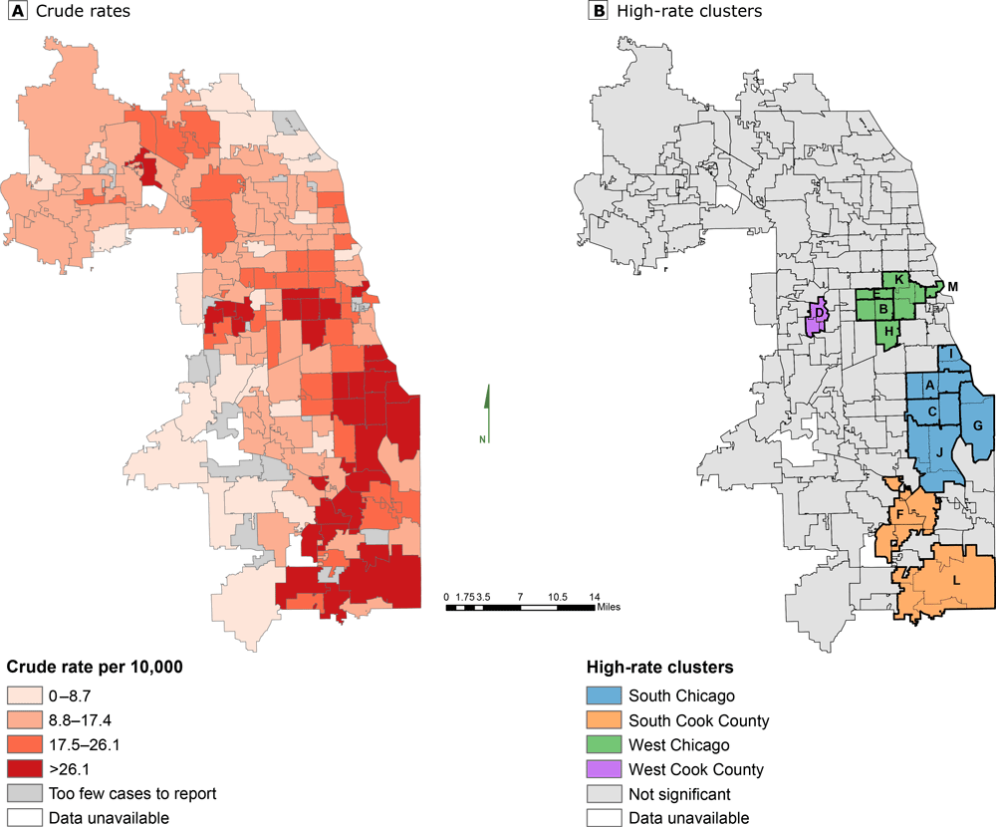
Asthma inpatient hospitalizations in Cook County, Illinois, by zip code, among children, adolescents, and young adults aged 0 to 19 years, 2011–2014. A, Crude rates. B, High-rate clusters, or neighborhoods with higher rates than would be expected under a constant rate hypothesis. Letters on map B correspond to clusters described in Table 1. These maps improve our understanding of rates of asthma inpatient hospitalization among young people in Cook County, Illinois, and will aid the Illinois Department of Public Health and asthma-focused community partners in identifying neighborhoods for asthma interventions. Data sources: Illinois Department of Public Health Division of Patient Safety and Quality and US Census Bureau (6,7).

## Background

Asthma is one of the top 5 principal diagnoses for inpatient hospitalizations among children and adolescents aged 1 to 17 years in the United States (1). Inpatient hospitalizations are used as a marker for severe asthma symptoms, suggesting poor management of disease and limited access to routine care (2). Preventing adverse childhood asthma outcomes is a priority of the Illinois Department of Public Health in collaboration with community and university partners (3). To understand the burden of asthma among young people in the greater Chicago (Cook County) area, we mapped crude rates of asthma inpatient hospitalizations among children, adolescents, and young adults aged 0 to 19 years, by zip code, in Cook County, Illinois, during 2011–2014. Additionally, we performed a cluster analysis to identify neighborhoods with high rates of asthma inpatient hospitalizations.

## Data Sources and Map Logistics

We obtained inpatient hospitalization records from the Illinois Department of Public Health Division of Patient Safety and Quality. To be included in our analysis, a patient record had to indicate the following: residence in Cook County, Illinois; age 0 to 19 years at hospital admission; discharge from January 1, 2011, through December 31, 2014; and a principal *International Classification of Diseases, Ninth Revision, Clinical Modification* (ICD-9-CM) diagnosis code of 493.XX (asthma) (4). We aggregated each record by zip code of residence after assessing that ZIP Code Tabulation Areas (5) and zip codes were well aligned for our data set. We obtained aggregated data for Cook County residents hospitalized out of state from the bordering states of Indiana, Iowa, Missouri, and Wisconsin, and we added these data to the final residential zip code counts.

We calculated the crude rate of annual asthma inpatient hospitalizations for each Cook County zip code by dividing the number of asthma inpatient hospitalizations during 2011–2014 by 4 times the 2014 American Community Survey population estimates for each zip code (ie, 4 years “at risk,” assuming a steady population size during 2011–2014) (6,7). We expressed rates as the number of asthma inpatient hospitalizations per 10,000 residents aged 0 to 19 years. We did not age-standardize rates because the proportion of the population aged 0 to 4 years and the proportion of the population aged 5 to 19 did not differ significantly by zip code. We created a map to illustrate the geographic distribution of crude rates by using Esri’s ArcMap version 10.4. For this map, we suppressed zip codes with fewer than 10 asthma inpatient hospitalizations during the 4-year study period. To inform the reference cutpoints in mapping the geographic distribution of hospitalization rates, we used Healthy People 2020 objective RD-2.2: 8.7 inpatient hospitalizations per 10,000 persons aged 5 to 64 (8). We created 4 categories of crude rate: 0 to 8.7, 8.8 to 17.4, 17.5 to 26.1, and more than 26.1 per 10,000.

We used SaTScan version 9.4.4, a free spatial scan statistics software (9), to identify clusters of zip codes with high rates of asthma inpatient hospitalizations. Briefly, scan statistics assess a constant rate hypothesis by using a scanning “window” in varying sizes, which moves across the study region (9,10). Of interest is whether the rate for the area inside the window is equal to the rate for the area outside the window. In our analysis, we looked for high-rate clusters, areas in which the number of asthma inpatient hospitalizations in a group of one or more zip codes (inside the window) was significantly greater than the number of asthma inpatient hospitalizations that would be expected if the hospitalization rates inside and outside the zip code group were equivalent. We used a Poisson distribution model and the Gini coefficient to identify the optimal cluster reporting size (9,10). After setting the maximum scanning window size at 50%, SaTScan found 3% to be the optimal maximum (Gini coefficient = 0.21) (11). We specified 999 Monte Carlo replications for the analysis. Output specification included all significant clusters (*P* < .05) ordered by log likelihood ratio (a measure of whether the rate inside the scanning window is higher than expected). We mapped all high-rate clusters in Esri’s ArcMap version 10.4. To maximize the utility of our study data for public health decision making, we ranked the clusters in 3 ways: by the observed number of inpatient hospitalizations; by the size of the population aged 0 to 19 years in each cluster; and by the relative excess rate of asthma inpatient hospitalizations, calculated as the observed number of inpatient hospitalizations divided by the expected number of inpatient hospitalizations.

## Highlights

We found 11,456 asthma inpatient hospitalizations among children, adolescents, and young adults aged 0 to 19 years in Cook County, Illinois, during 2011–2014. Zip code–specific rates ranged from 4.0 to 65.8 hospitalizations per 10,000 persons aged 0 to 19 years with the highest rates 2 to 3 times greater than the Cook County rate of 20.0. Of 174 zip codes in the study area, 31 had a crude hospitalization rate of more than 26.1 per 10,000 persons aged 0 to 19 years (Map A). 

We identified 13 high-rate clusters of asthma inpatient hospitalizations (Map B and Table 1). These clusters were located primarily in the western and southern areas of Chicago and southern Cook County. Together, the zip codes in these 13 high-rate clusters comprised 44.9% (5,142 of 11,456) of asthma inpatient hospitalizations and 25.4% (1,459,949 of 5,741,061) of young people aged 0 to 19 years in Cook County. The degree of precision in relative excess rate estimates varied because of differences in the population size and the number of asthma inpatient hospitalizations in each cluster. During the 4-year study period, cluster A had the largest number of inpatient hospitalizations (n = 704), cluster M had the smallest total population (n = 18,317), and cluster D had the highest relative excess rate (2.78 [95% confidence interval, 2.38–3.18] (Tables 1 and 2).

**Table 1:** High-Rate Clusters of Asthma Inpatient Hospitalizations Among Children, Adolescents, and Young Adults Aged 0–19 Residing in Cook County, Illinois, 2011–2014^a^

Clusters	Zip Code of Patient’s Residence	Total No. of Asthma Inpatient Hospitalizations	Total Population^b^	Rate per 10,000^c^	Relative Excess Rate^d^ (95% CI)
Cook County	—	11,456	5,741,061	20.0	—
Cluster A	60621, 60636, 60637	704	155,918	45.1	2.26 (2.10–2.43)
Cluster B	60624, 60644	525	122,391	42.8	2.15 (1.97–2.33)
Cluster C	60619, 60620	573	145,564	39.3	1.97 (1.81–2.13)
Cluster D	60141, 60153, 60155	187	33,663	55.4	2.78 (2.38–3.18)
Cluster E	60651	311	75,076	41.3	2.08 (1.85–2.31)
Cluster F	60426, 60428, 60429, 60469, 60472, 60478	379	103,180	36.7	1.84 (1.66–2.03)
Cluster G	60617, 60649	464	144,397	32.1	1.61 (1.46–1.76)
Cluster H	60623	393	124,727	31.4	1.58 (1.42–1.74)
Cluster I	60615, 60653	234	71,882	32.5	1.63 (1.42–1.84)
Cluster J	60628, 60643, 60827	468	168,074	27.8	1.40 (1.27–1.52)
Cluster K	60612, 60622, 60642, 60647	476	172,150	27.6	1.39 (1.26–1.51)
Cluster L	60411, 60425, 60461, 60466, 60475	360	124,610	28.8	1.45 (1.30–1.60)
Cluster M	60610	68	18,317	37.1	1.86 (1.42–2.30)

Abbreviation: CI, confidence interval.

a Data source: Illinois Department of Public Health Department of Patient Safety and Quality. All high-rate clusters were identified by using SaTScan version 9.4.4 (9) and a significance level of *P* <.05. Clusters are ordered by log likelihood ratio, from highest to lowest. For each scanning window, we calculated the log of the likelihood ratio. The likelihood ratio is the likelihood under the alternative hypothesis (the rate inside the scanning window is greater than the rate outside the scanning window) divided by the likelihood under the null hypothesis (rate inside scanning window is equivalent to rate outside scanning window).

b Calculated as 4 years × the number of residents aged 0–19 years in 2014.

c Calculated as the number of asthma inpatient hospitalizations per 10,000 residents aged 0–19 years.

d Calculated as the number of observed asthma inpatient hospitalizations in each cluster divided by the expected number of asthma inpatient hospitalizations in each cluster.

**Table 2:** High-Rate Clusters of Asthma Inpatient Hospitalizations Among Children, Adolescents, and Young Adults Aged 0–19 Years Residing in Cook County, Illinois, Ranked by Total Number of Asthma Inpatient Hospitalizations, Total Population, and Relative Excess Rate, 2011–2014^a^

Rank	Total No. of Asthma Inpatient Hospitalizations^b^	Total Population^c^	Relative Excess Rate^d^
1	A	M	D
2	C	D	A
3	B	I	B
4	K	E	E
5	J	F	C
6	G	B	F
7	H	L	M
8	F	H	G
9	L	G	I
10	E	C	H
11	I	A	L
12	D	J	J
13	M	K	K

a Data source: Illinois Department of Public Health Department of Patient Safety and Quality. All high-rate clusters were identified by using SaTScan version 9.4.4 (9) and a significance level of *P* <.05. Rates were calculated as the number of asthma inpatient hospitalizations per 10,000 residents aged 0–19 years. All letters indicate clusters. Cluster locations are illustrated in Map B. Cluster zip codes: A = 60621, 60636, 60637; B = 60624, 60644; C = 60619, 60620; D = 60141, 60153, 60155; E = 60651; F = 60426, 60428, 60429, 60469, 60472, 60478; G = 60617, 60649; H = 60623; I = 60615, 60653; J = 60628, 60643, 60827; K = 60612, 60622, 60642, 60647; L = 60411, 60425, 60461, 60466, 60475; M = 60610.

b Ranked from largest to smallest.

c Ranked from smallest to largest. Calculated as 4 years × the number of residents aged 0–19 years in 2014.

d Ranked from highest to lowest. Calculated as the number of observed asthma inpatient hospitalizations in each cluster divided by the expected number of asthma inpatient hospitalizations in each cluster.

## Action

We identified substantial variation in the number of asthma inpatient hospitalizations by zip code in Cook County. The grouping of zip code clusters by neighborhood will be useful to organizations working in these neighborhoods. Our ranking of asthma clusters can be used to examine zip codes in which interventions can be introduced or accelerated. The ranking by relative excess rate may be most useful to organizations concerned with geographic disparities in asthma inpatient hospitalization rates, whereas data on total number of asthma inpatient hospitalizations may be most useful to organizations interested in programs or policies to reduce the absolute numbers of young people with asthma. Alternatively, the ranking by total size of the population aged 0 to 19 years may be most relevant when resources are limited or when introducing resource-intensive care coordination programs. These priorities can be combined to best address the needs and interests of the community.

One limitation of this ranking framework is the inability to assess the unique drivers of risk in each cluster; each set of unique drivers may require its own uniquely designed intervention. Assessment of the modifiable characteristics of each cluster may lead to targeted health interventions. Other limitations were an inability to identify hospital readmissions and an inability to detect false-positive clusters caused by repeated significance testing. False positive concerns are often addressed by considering only the 2 most likely clusters. However, excluding information on all potential clusters may not align with public health priorities. 

Our study adds a framework for combining public health decision-making considerations and exploratory spatial statistical tools. We will continue to investigate the application and value of cluster analysis ranking to inform asthma programs for young people in Illinois.
